# The Impact of Martial Arts-Based Fall Training Programs on Health and Well-Being in Individuals with Disabilities: A Systematic Review

**DOI:** 10.3390/healthcare14162644

**Published:** 2026-08-20

**Authors:** Hugo Ângelo, Alain Massart, Jorge Abrantes, Hugo Sarmento, Maria João Campos, José Pedro Ferreira

**Affiliations:** 1 Faculty of Sport Sciences and Physical Education, University of Coimbra, 3040-248 Coimbra, Portugal; uc2023138227@student.uc.pt; 2 Interdisciplinary Center for the Study of Human Performance (CIPER), Faculty of Sport Sciences and Physical Education, University of Coimbra, 3040-248 Coimbra, Portugal; alainmassart@fcdef.uc.pt (A.M.); mjcampos@fcdef.uc.pt (M.J.C.);

**Keywords:** martial arts, judo, safe falls, intellectual and developmental disabilities, injury prevention, balance

## Abstract

**Background/Objectives**: Regular participation in physical activity is essential for maintaining health and well-being and plays a key role in disease prevention and quality of life, both in the general population and among people with disabilities, who represent approximately 16% of the global population. Martial arts-based physical activity programs incorporating safe-falling techniques may contribute to improving physical fitness, reducing fall-related injuries, and supporting adherence to the World Health Organization physical activity recommendations. This systematic review primarily aimed to identify martial arts-based fall-training programs, particularly judo-based interventions, and examine their effects on health and well-being in individuals with intellectual disability. As a secondary objective, evidence from other populations and martial arts disciplines was considered to identify relevant fall-training protocols and transferability. **Methods**: The research question was formulated using the PEO framework. Eligibility criteria included: people with intellectual and developmental disabilities or special educational needs; exposure to exercise programs involving martial arts, combat sports, or judo; and outcomes related to safe falling, falls, balance, motor skills, and physical condition. A systematic literature search was conducted between December 2025 and April 2026 on the Web of Science Core Collection, Scopus, PubMed, and SPORTDiscus in accordance with PRISMA guidelines. **Results**: From a total of 658 records identified, 27 studies met the inclusion criteria, encompassing 1892 participants with various disabilities, including intellectual disability, autism spectrum disorder, visual impairment, and deafness. The interventions involved different martial arts modalities, such as judo, taekwondo, karate, tai chi, and capoeira. Overall, the available evidence suggests beneficial effects on physical fitness, motor abilities, balance, psychosocial outcomes, and safe-falling skills across different populations; however, substantial clinical and methodological heterogeneity was observed among the included studies. **Conclusions**: No studies investigating structured martial arts-based fall-training programs in individuals with intellectual disability were identified. Although evidence from other populations is promising, it remains heterogeneous. Further high-quality studies are needed to evaluate these interventions in individuals with intellectual disabilities.

## 1. Introduction

Regular physical activity is essential for maintaining and improving the health and well-being of all individuals. The World Health Organization (WHO) recognizes physical activity as an essential tool for promoting physical and mental health, preventing disease, and improving quality of life, and since 2020, its recommendations have explicitly included individuals with disabilities [1,2]. According to the WHO, approximately 1.3 billion people live with a significant disability, representing around 16% of the global population (i.e., one in every six individuals).

Falls have been reported as a leading cause of non-fatal injuries treated in hospital settings across almost all age groups [3]. Individuals with intellectual disabilities often exhibit impaired musculoskeletal function, including impaired balance and early onset of sarcopenia. These impairments are associated with reduced independence in activities of daily living, lower levels of autonomy, an increased risk of falls [4,5], poorer physical fitness [6], and more sedentary lifestyles [7]. Furthermore, fall-related functional impairment increases healthcare costs because of the need to treat both immediate injuries and long-term disability [8]. Even non-injurious falls may have disabling consequences and have been associated with activity restriction, social isolation, deconditioning, increased fear of falling, and depression [8]. When combined with factors such as multimorbidity and polypharmacy, which are common among individuals with neurodevelopmental disorders, these impairments may substantially increase the risk of falls and accelerate age-related functional decline [9].

In judo, as in other martial arts that incorporate throwing techniques, the acquisition of safe-falling techniques is a fundamental component of training. These techniques are intended to modify instinctive protective responses that may increase the risk of head and upper-limb injuries during an unexpected fall. Therefore, regular participation in these disciplines not only contributes to meeting the World Health Organization (WHO) recommendations for physical activity but may also represent a promising strategy for reducing fall-related injuries.

Previous reviews have consistently reported that judo-based interventions provide a wide range of health-related benefits across different populations. In individuals with neurodevelopmental disorders, judo practice has been associated with improvements in physical activity levels, social interactions, emotional well-being, and cognitive functioning, supporting its potential as a complementary therapeutic and educational approach [10]. Similarly, judo-based exercise programs have demonstrated positive effects on health-related outcomes in middle-aged and older adults with no previous judo experience, suggesting that this martial art can be safely adapted to promote healthy ageing [11]. In children, judo training has also been linked to multiple physical health benefits and has been identified as a valuable strategy for promoting regular physical activity in line with World Health Organization recommendations [12]. Taken together, these findings indicate that judo-based interventions are associated with broad physical, psychological, cognitive, and social benefits across diverse populations.

More recent evidence has further strengthened the potential of adapted martial arts as a strategy to promote health and well-being in individuals with disabilities. An adapted capoeira intervention significantly improved the psychomotor profile of individuals with intellectual disability, particularly in domains related to balance, body awareness, and motor coordination [13]. More recently, an adapted judo program significantly improved physical fitness components, including strength, balance, and flexibility, while also enhancing psychosocial outcomes such as self-esteem and social interaction in adolescents with Down syndrome [14]. These findings reinforce the growing interest in adapted martial arts as inclusive interventions capable of promoting multidimensional health benefits in populations with disabilities.

Despite these encouraging findings, important gaps remain in the literature. Previous reviews have primarily focused on the general health benefits of judo practice or judo-based exercise programs, without specifically examining interventions designed to teach safe falling skills. Moreover, no previous review has synthesized the evidence regarding martial arts-based fall training programs for individuals with Intellectual Disability (ID), a population that may particularly benefit from interventions aimed at improving safe landing strategies, motor competence, and injury prevention.

Given the absence of reviews addressing this specific topic, a systematic synthesis of the available evidence is warranted. A systematic review is therefore needed to determine whether structured judo-based fall training programs have been implemented in individuals with ID and to evaluate their effects on health and well-being outcomes. Because research in this field is still limited, it is also important to identify evidence from similar fall-training interventions based on other martial arts, as well as studies evaluating judo fall-training protocols in other populations. This broader perspective may provide valuable insights into the transferability of these interventions and inform future research.

Unlike previous reviews, which have examined the general effects of judo practice across different populations, the present review specifically focuses on martial arts-based interventions in which the teaching and practice of safe falling constitute a core component of the intervention.

In this review, individuals with intellectual disability (ID) were considered the primary population of interest. Evidence from other populations exposed to structured martial arts-based fall-training programs was considered as a secondary or complementary source of evidence, primarily to assess the potential transferability of findings and to identify relevant fall-training protocols that may inform future research in individuals with intellectual disability.

## 2. Materials and Methods

The systematic review was conducted in accordance with the Cochrane guidelines [15] and was carried out according to the PRISMA system guidelines [16]. This study was registered on the OSF platform on 16 December 2025 (Registration https://doi.org/10.17605/OSF.IO/WF5SV).

### 2.1. Search Strategy and Data Source

The literature search for this systematic review was conducted from December 2025 to April 2026 by two authors (Hugo Ângelo [HA] and Jorge Abrantes [JA]) using the following electronic databases: Web of Science Core Collection, Scopus, PubMed, and SPORTDiscus. In addition, the reference lists of all eligible full-text articles were manually screened to identify further relevant studies. Grey literature and relevant expert documents were also considered when appropriate.

The research question was formulated according to the PEO (Population, Exposure, Outcome) framework: Population—individuals with disabilities or special educational needs; Exposure—exercise programs, martial arts, physical activity, combat sports, and judo; Outcome—safe-falling skills, falls, balance, motor skills, physical fitness, and injury prevention.

The search strategy was designed to prioritize judo, given its extensive use of throwing techniques and the consequent emphasis placed on the teaching and practice of safe-falling techniques. The broader concept of martial arts was also retained to characterize the exposure of interest and to identify potentially relevant evidence beyond judo. Because the primary search was specifically oriented towards judo, complementary screening of reference lists was subsequently undertaken to identify additional studies involving other martial arts.

The following search strategy was applied:

(“intellectual disabilit” OR “development* disabilit*” OR “people with disabilit*” OR “special education*” OR “special educational need*”) AND (“judo”) AND (“exercise* program*” OR “martial art*” OR “physical activit*” OR “combat sport*”) AND (“safe-fall*” OR “fall*” OR “balance” OR “motor skill*” OR “physical condition” OR “injur*” OR “health”).

Search terms were identified based on terminology previously used in the titles, abstracts, and keywords of relevant publications. The search strategy was revised to ensure the correct use of Boolean operators, quotation marks, truncation symbols, and spacing, while preserving consistency with the search protocol registered in the Open Science Framework (OSF). Complete database-specific search strategies and applied filters are provided in Supplementary Table S1 to facilitate transparency and reproducibility.

### 2.2. Selection of Studies and Eligibility Criteria

For the identification of potentially eligible studies, initial selection was performed by two independent authors (HA and JA) based on title and abstract. Disagreements between the authors were resolved by consensus, and if necessary, a third independent reviewer (AM) was consulted. For this study, the full texts were read by only one author (HA) to verify compliance with eligibility criteria, and if necessary, a second independent author (JA) was consulted. Only publications and abstracts in English were considered, and no filters were applied regarding the year of publication.

Eligibility criteria included: According to the pre-registered PEO framework, studies were eligible for inclusion if they met the following criteria: (1) Population: people with intellectual and developmental disabilities or special educational needs; (2) Exposure: participation in exercise programs involving martial arts, physical activity, combat sports, or judo; and (3) Outcomes: quantitative metrics related to safe falling, fall incidence, balance, motor skills, and physical condition. Within this eligibility framework, individuals with intellectual disability (ID) were considered the primary population of interest, whereas evidence from other eligible populations was considered secondary or complementary, primarily to assess the potential transferability of findings and to identify relevant fall-training protocols.

Regarding Study Design, eligible records were restricted to quantitative interventional studies to evaluate the intervention outcomes effectively. Specifically, the included study designs comprised: randomized controlled trials (RCTs, including parallel and crossover designs), quasi-experimental studies (e.g., non-randomized controlled trials with a control group), non-randomized studies, pre-post intervention studies (single-group pre-test/post-test designs), and pilot interventional studies reporting quantitative outcome measurements.

Exclusion criteria: Observational studies without an active intervention (e.g., cross-sectional, cohort, or case-control studies), qualitative-only studies, case reports or case series, narrative reviews, systematic or scoping reviews, conference abstracts without full text, and book chapters were excluded.

### 2.3. Data Extraction

In this study, one author (HA) extracted information from eligible studies into a standardized Excel spreadsheet. The main extracted data categories included: author(s) and publication year, sample size and participant characteristics, martial arts modality, fall prevention or safe-landing instruction methodology, variables studied, and main findings. To ensure data accuracy and minimize transcription errors, the extracted data were double-checked against the original articles by the same primary author, and a second author (JA) independently spot-checked a random sample of the extracted records and was consulted in case of ambiguous data reporting.

### 2.4. Variables

This systematic review studied articles that reported the exposure of populations with intellectual disabilities to physical activity programs involving martial arts or combat sports and verify its effects on the following variables: Motor Abilities, Balance, Mental Health, Well-being, Physical Condition, Biochemical Factors, and Falls Quality.

### 2.5. Quality Assessment

To evaluate the methodological quality and risk of bias of the included studies, we employed the “Tool for the assEssment of Study qualiTy and reporting in EXercise” (TESTEX) [17].

## 3. Results

### 3.1. Study Selection

**Figure 1:**
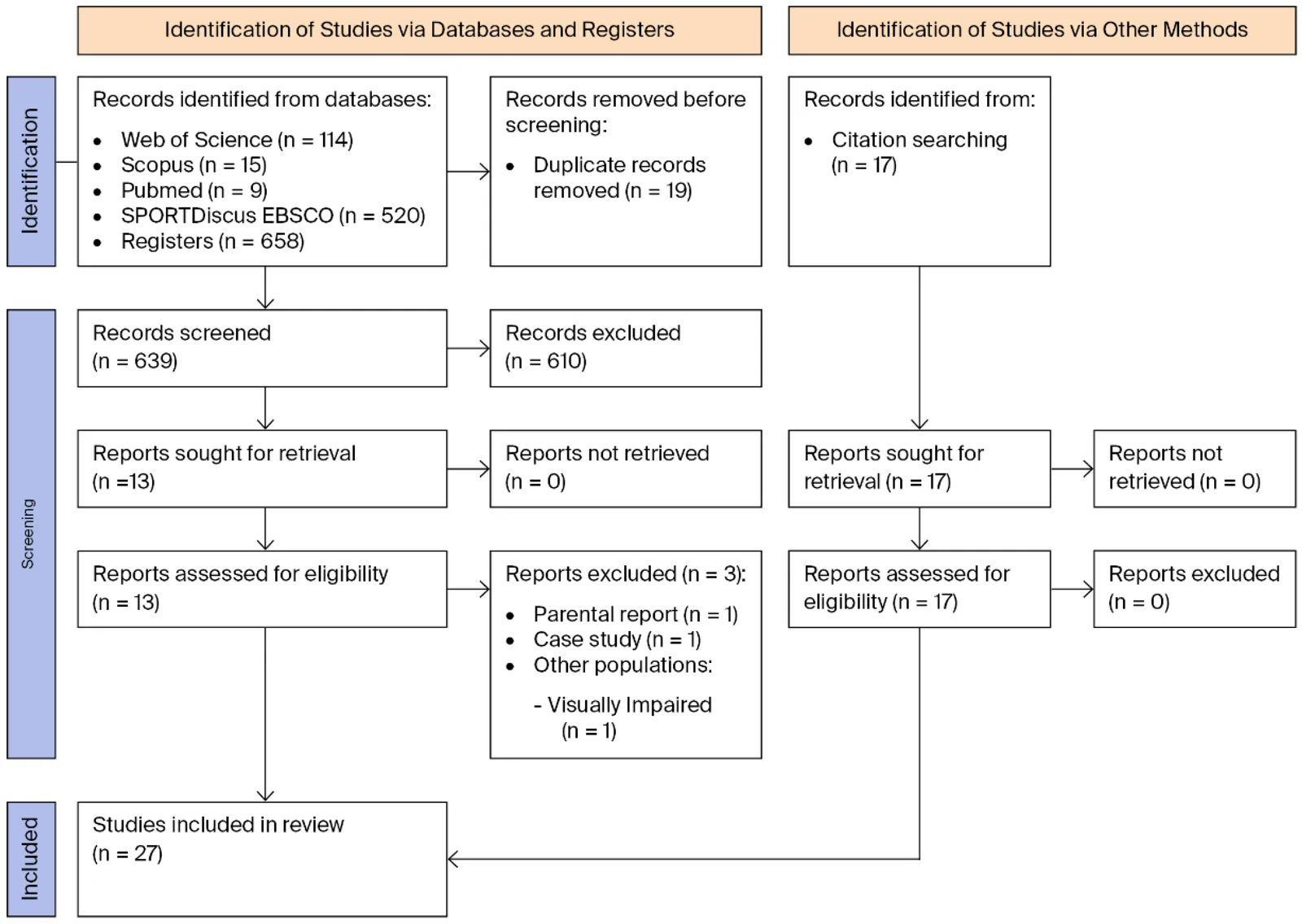
Identification of studies via databases and registries. Adapted from [15].

A total of 658 publications were identified through the bibliographic resources explored in the initial search, and a total of 17 studies were identified from citation searching. After eliminating duplicate studies and applying the eligibility criteria, 10 studies were identified via databases (at the end of the screening process, 3 studies appeared to meet the inclusion criteria but were excluded due to their characteristics—population or methodology); a total of 27 articles were selected for this systematic review. Figure 1 displays a detailed overview of the selection process.

### 3.2. Methodological Quality Assessment

The TESTEX [17] scale score for each study included in the review is shown in Table 1. We selected the TESTEX scale because it is a reliable tool, specific to exercise scientists, that facilitates a comprehensive review of exercise training trials.

### 3.3. Characteristics of the Studies Included in the Review

The main characteristics of the included studies are summarized in Table 2. The primary population of interest in this review was individuals with intellectual disability (ID). Fourteen studies investigated martial arts interventions in this population [13,14,18,25,27,28,30,33,34,35,36,37,38,39]. The remaining studies involved other populations or health conditions and were considered as complementary evidence to assess the potential transferability of martial arts-based interventions and to identify structured fall-training protocols relevant to the primary research question.

Among the 27 included studies, judo was the most frequently investigated martial art (*n* = 14), followed by tai chi (*n* = 3), karate (*n* = 3), capoeira (*n* = 2), and multisport interventions incorporating martial arts (*n* = 2). Taekwondo, an oriental martial arts system, and traditional Chinese Chi Kung were each represented in one study.

Five studies specifically evaluated structured judo-based fall-training programs, including Judo4Balance (*n* = 3) [8,9,19] and Safe Fall (*n* = 2) [23,40].

Intervention duration ranged from 5 to 48 weeks, while training frequency varied between one and three sessions per week. The studies employed different methodological designs, including randomized controlled trials, non-randomized controlled studies, quasi-experimental studies, pre–post intervention studies, and one study protocol.

A detailed description of the methodological characteristics of each included study, including participants, intervention protocols, variables analyzed and principal findings, is presented in Table 3.

Detailed characteristics of the structured judo-based fall-training programs identified in the included studies are presented in Supplementary Table S2.

To facilitate the interpretation of the evidence, the main findings were also synthesized according to the primary outcomes evaluated across the included studies (Table 4).

The outcome measures reported across the studies were grouped into seven categories: motor abilities, balance, mental health, well-being, physical fitness, biochemical markers, and fall quality. Motor abilities (*n* = 16) and balance (*n* = 11) were the most frequently investigated outcomes.

### 3.4. Effects of the Intervention

The primary emphasis in this systematic review is placed on individuals with Intellectual Disability (ID) and we started by analyzing studies that presented the positive influence of martial arts programs on participants with intellectual disabilities. The effects of different martial arts programs have demonstrated a significant impact on various categories of variables associated with the health and well-being of participants with intellectual disabilities, such as motor abilities [13,14,18,27,33,37], balance [13,14,39], mental health [27], well-being [14,36,37], physical fitness [14,39] and biochemical factors [18].

Research in this field is still limited, and it was also important to identify evidence from studies that consider other neurodevelopmental disorders populations such as autism spectrum disorder and it was also possible to observe positive influence of martial arts programs in participants on several of categories of variables we intended to study as motor abilities [25,34,35,38], balance [30], mental health [28,35], well-being [28,34] and physical fitness [38].

As expected, one of the main outcome variables, fall quality, was not considered in studies that specifically examine interventions designed to teach safe falling skills in neurodevelopmental disorder populations. We only had some references to the effect of the improvement in agility observed and its relevance to better functional mobility and fall prevention [14], but still no fall quality evaluation.

Therefore, looking to provide valuable insights into the transferability of these interventions, we expanded the scope of this review to include studies involving other populations exposed to martial arts-based interventions. Specifically, we analyzed studies investigating Tai Chi programs in individuals with multiple sclerosis, which reported benefits for motor abilities, balance and mental health [20]; Tai Chi interventions in individuals with rheumatoid arthritis, which demonstrated improvements in mental health and well-being [24]; and Tai Chi programs for older adults, which showed positive effects on well-being [29]. We also reviewed karate interventions for children with developmental coordination disorder, reporting benefits in motor abilities, balance, and physical fitness [26]; qigong (Chi Kung) interventions for individuals with Parkinson’s disease, which improved motor function [31]; and capoeira programs for deaf children, which generated positive effects on balance [32]. In line with this broader perspective, we also considered two studies involving multisport methodologies. The first, conducted in individuals with autism spectrum disorder and intellectual disability, did not report differences between groups in motor skills and physical fitness [21]. The second compared two exercise interventions—sports participation (Goalball, football, and martial arts) and functional training (bodyweight exercises)—in adults with visual impairment and demonstrated benefits in well-being and physical fitness [22]. Finally, we were able to identify two fall-prevention programs: “Judo4Balance”, reported in three studies [8,9,19], and “Safe Fall”, reported in two studies [23,40].

“Judo4Balance” program was a judo protocol tested in adult workers and demonstrated a significant impact on variables such as balance, physical fitness and fall quality [8,19]; and in older adults with impact on balance, well-being, physical fitness and fall quality [9]. “Safe Fall” was also a judo protocol applied to school-age children and presented benefits on balance and fall quality [23], and when applied to adolescents, demonstrated positive impact on motor abilities, balance and fall quality [40]. Therefore, in these five studies [8,9,19,23,40] we were able to identify the positive effects associated with a martial arts program (judo).

## 4. Discussion

This systematic review provided a comprehensive analysis of the exposure of populations with intellectual disability (ID) to physical activity and exercise programs involving martial arts or combat sports, evaluating their potential effects on motor abilities, balance, mental health, physical fitness, biochemical markers, and fall quality. Because the literature directly evaluating individuals with pure ID exposed to combat sports and specialized fall-training protocols remains limited (particularly regarding fall quality), the scope of this review was broadened to encompass related clinical populations (e.g., autism spectrum disorder, physical/sensory impairments, older adults) as well as adapted physical activity specialists and healthcare providers in practical settings. Synthesizing evidence across these diverse settings was essential to map transferable intervention principles and to evaluate the feasibility of implementation in adapted exercise contexts.

### 4.1. The Effects of the Multiple Protocols of Martial Arts on Health and Well-Being

The studies included in this review encompassed a wide range of martial arts-based interventions, including judo, karate, Tai Chi, Qigong, Capoeira, and specialized fall-training programs such as Judo4Balance and Safe Fall. Rather than considering these interventions in isolation, the positive health and functional outcomes observed across disciplines can be largely explained by common underlying neurophysiological, biomechanical, and psychosocial mechanisms.

#### Mechanisms Driving Functional and Physiological Adaptations

The positive effects observed across different martial arts may be explained by common characteristics shared by these disciplines, including repetitive practice of complex motor skills, dynamic postural control, proprioceptive stimulation, and progressive improvements in neuromuscular coordination.

- Motor Abilities: Improvements in gross and fine motor skills—observed following Judo, Karate, and Capoeira interventions [13,18,27,37]—are mediated by repetitive motor practice, task-specific learning, motor planning, and enhanced sensorimotor integration. The continuous execution of complex, multi-joint movement patterns forces the central nervous system to refine motor control strategies and dynamic movement execution.
- Balance and Postural Control: Gains in balance across diverse populations (e.g., via Tai Chi, Capoeira, Judo4Balance, and Oriental Struggles) [8,9,13,20,32,39] stem from constant proprioceptive stimulation, dynamic center-of-mass perturbations, dynamic stability demands, and vestibular adaptations induced by weight shifts, single-leg stances, and multi-directional footwork.
- Psychosocial Well-being and Mental Health: Reductions in anxiety/depressive symptoms and enhancements in self-esteem, self-confidence, and social interaction [14,20,24,27,36] are underpinned by increased self-efficacy, structured peer interaction, mastery experiences gained through skill acquisition, and the supportive social climate intrinsic to martial arts dojos.
- Fall Quality and Safety: Improvements in fall safety observed in Judo4Balance and Safe Fall protocols [8,9,19,23,40] are driven by repeated breakfall practice (ukemi), which leads to motor automatization of protective reactions (e.g., tucking the chin, dissipating impact force across larger surface areas), reduced fear of falling, and improved protective responses during sudden loss of balance.

Biochemical responses also reflect these physiological adaptations. For instance, judo training in Down syndrome [18] induced antioxidant enzymatic adaptations (SOD and catalase activity) alongside exercise-induced oxidative stress (TBARS and lipid peroxides), emphasizing that exercise dosage and intensity must be carefully tailored to account for heightened susceptibility to oxidative damage in specific developmental conditions.

### 4.2. Comparison with Previous Literature and Systematic Reviews

Our findings are consistent with those reported by Descamps et al. [10], who concluded that adapted judo promotes physical activity, social participation, and emotional well-being among individuals with neurodevelopmental disorders. Similarly, Chan et al. [11] observed beneficial effects of judo-based exercise programs in middle-aged and older adults, while Kowalczyk et al. [12] highlighted the positive health effects of judo practice in school-aged children.

However, unlike previous reviews, the present review specifically investigated structured judo-based fall-training programs and demonstrated that no such interventions have yet been evaluated in individuals with intellectual disability.

### 4.3. Methodological Considerations and Heterogeneity

Considering this review, a significant heterogeneity regarding martial arts programs is evident, alongside a consensus on their physical and psychological benefits. However, detailed reporting of intervention characteristics is often missing, particularly regarding relative exercise intensity assessment and energy expenditure. Another major methodological limitation across many primary studies is the lack of a control group (present in only 16 of the 27 included studies).

### 4.4. Limitations of the Study

Several limitations of this review should be acknowledged. First, no studies were identified investigating structured martial arts-based fall-training programs specifically designed for individuals with intellectual disability. Consequently, the review necessarily included evidence from other disability groups and populations to explore the potential transferability of these interventions.

Second, the included studies presented high clinical and methodological heterogeneity, characterized by small sample sizes, varying intervention protocols (duration, frequency, and type of martial art), and diverse outcome measures. The possibility of publication bias cannot be excluded, as the small number and heterogeneity of the included studies precluded a formal assessment of publication bias. Therefore, the available evidence may be subject to selective publication or reporting of positive findings.

Third, only English-language studies were included, which may have omitted relevant non-English literature.

Fourth, the GRADE approach was not formally applied to rate the overall certainty of evidence. This decision was due to the marked clinical and methodological heterogeneity of the included studies, which precluded a meta-analysis and limited the objective evaluation of key statistical GRADE domains, such as imprecision and inconsistency. Nonetheless, the methodological quality and reporting of individual studies were rigorously evaluated using the TESTEX scale, a validated tool specifically designed for exercise training interventions.

Finally, a methodological limitation concerns the selection and data extraction processes: while initial title and abstract screening was performed independently by two authors, full-text eligibility verification and data extraction were conducted primarily by one author, with a second author consulted in cases of uncertainty or for spot-checking.

Although PRISMA 2020 recommends independent dual-reviewer assessment during study selection and data extraction whenever feasible, resource constraints dictated this single-reviewer approach. To mitigate potential reporting bias, predefined eligibility criteria and standardized extraction forms were strictly applied, and borderline or complex data points were thoroughly discussed with senior team members.

### 4.5. Implications for Future Research

Analyzing the intervention protocols suggests that a 12- to 14-week martial arts program (with appropriate frequency and progression) is adequate to elicit meaningful adaptations in motor control, balance, and physical fitness.

Judo, due to its specialized throwing techniques and inherent emphasis on fall techniques (ukemi), represents the most suitable martial art discipline to adapt for fall-prevention projects. Given that limitations in motor control, postural stability, and adaptive movement responses increase vulnerability to fall-related injuries in populations with ID, developing judo-based fall safety interventions represents a critical preventive strategy.

From an applied perspective, the findings of this review provide actionable insights across multidisciplinary healthcare and exercise settings. For physical therapists, the sensorimotor, proprioceptive, and dynamic postural demands of martial arts—such as multi-directional footwork and weight shifts—offer novel functional strategies to address dynamic stability and gait impairments. Rehabilitation specialists can integrate structured breakfall protocols (ukemi) into therapeutic regimens to promote the motor automatization of protective responses, thereby equipping individuals with motor strategies to dissipate impact forces and reduce severe injury risk during accidental falls. Furthermore, adapted physical activity specialists can utilize these task-oriented, structured sports to enhance motor planning, coordination, and physical fitness while fostering social inclusion and self-efficacy in community and educational frameworks. Finally, healthcare providers in clinical settings can advocate for adapted martial arts and fall prevention programs as multi-systemic, non-pharmacological interventions that concurrently improve physical health, motor autonomy, psychosocial well-being, and overall quality of life in populations with intellectual disabilities.

Although direct evidence is currently unavailable for individuals with intellectual disability, the positive effects observed in other populations suggest that structured judo-based fall-training programs could represent a promising strategy for adapted physical activity, rehabilitation, special education, and community-based fall-prevention programs. Future studies should evaluate the feasibility, safety, and effectiveness of these interventions in individuals with intellectual and developmental disabilities.

### 4.6. New Lines of Research

Despite the heterogeneity of the martial arts interventions and the populations included, this review enabled the identification of studies investigating structured martial arts-based fall training programs and, more specifically, whether judo-based fall training interventions have been implemented in individuals with intellectual disability (ID). The available evidence also allowed the evaluation of their potential effects on health- and well-being-related outcomes, while providing valuable insights into the transferability of these interventions across different populations.

However, the findings highlight a substantial gap in the literature. Very few, or no, studies have specifically investigated structured judo-based fall training programs in individuals with ID. Consequently, future research should prioritize the development and evaluation of standardized judo-based fall training protocols tailored to this population. Such studies should investigate their effects on health and well-being, including physical fitness, motor abilities, balance, fall quality and safety, confidence in falling safely, and injury prevention. In addition, well-designed randomized controlled trials with larger sample sizes and long-term follow-up are needed to establish the effectiveness of these interventions and to support their implementation in educational, rehabilitation, and community settings.

## 5. Conclusions

This systematic review identified no studies investigating structured martial arts-based fall-training programs, particularly judo-based fall training, in individuals with intellectual disability (ID). Consequently, direct evidence regarding the effects of these interventions on health, well-being, and fall-related outcomes in this population remains unavailable.

Nevertheless, the available evidence suggests that participation in martial arts, including judo, karate, taekwondo, tai chi, and capoeira, is associated with beneficial effects on physical fitness, motor abilities, balance, mental health, well-being, biochemical markers, and fall-related outcomes across different populations. These findings suggest that martial arts-based interventions, particularly those incorporating safe-falling techniques, may improve health and functional outcomes; however, they should be interpreted with caution given the clinical and methodological heterogeneity of the available evidence.

Although direct evidence in individuals with ID is unavailable, findings from other martial arts interventions and populations provide preliminary evidence that may inform future research. Developing and evaluating standardized judo-based fall-training programs for individuals with ID should be considered a research priority to determine whether these interventions can improve health, well-being, safe-falling skills, and contribute to injury prevention in this population.

## Figures and Tables

**Table 1:** Quality assessment of the TESTEX scale.

Study	1.	2.	3.	4.	5.	6.			7.	8.		9.	10.	11.	12.	Total
						6.1	6.2	6.3		8.1	8.2					
Aguiar Jr, A. S., et al. (2008) [18]	1	0	0	0	0	1	1	1	0	0	0	1	0	1	1	7
Arkkukangas, M., et al. (2020) [8]	1	0	0	0	0	1	1	1	0	0	0	1	0	1	0	6
Arkkukangas, M., et al. (2021) [19]	1	1	0	1	0	1	0	1	0	1	1	1	1	1	0	10
Arkkukangas, M., et al. (2022) [9]	1	1	1	1	0	1	0	1	0	1	1	1	1	1	0	11
Burschka, J. M., et al. (2014) [20]	1	0	0	1	0	1	0	1	0	1	1	0	0	1	0	7
Cha, J. Y., et al. (2020) [21]	1	0	0	1	0	1	1	1	0	0	0	0	0	1	1	7
da Silva, R. B. P., et al. (2022) [22]	1	1	0	1	0	1	0	1	0	1	1	0	1	1	0	9
DelCastillo-Andrés, O., et al. (2019) [23]	1	1	1	1	0	1	0	1	0	0	0	1	1	1	0	9
Du, J., et al. (2022) [24]	1	1	1	1	0	1	0	1	0	1	1	1	1	1	0	11
Garcia, J. M., et al. (2020) [25]	1	0	0	1	0	1	0	1	0	0	0	1	0	1	0	6
Ghadiri et al. (2022) [26]	1	0	0	1	0	0	0	1	0	1	1	1	0	1	0	7
Gleser, J. M., et al. (1992) [27]	1	0	0	0	0	1	0	0	0	0	0	1	0	1	0	4
Greco & De Ronzi (2020) [28]	1	0	1	1	1	1	0	1	0	1	1	1	0	1	0	10
Greenspan, A. I., et al. (2007) [29]	1	1	0	1	0	0	0	1	1	0	0	1	1	0	0	7
Kim, Y., et al. (2016) [30]	1	0	0	0	0	1	0	1	0	1	1	1	1	1	0	8
Li, Z. L., et al. (2019) [31]	1	1	1	1	1	1	0	1	1	1	1	1	1	1	1	14
Lima, R. (2017) [32]	1	1	0	1	0	1	0	0	0	1	0	0	0	0	0	5
Maslesa, S., et al. (2012) [33]	1	0	0	0	0	1	0	1	0	0	0	1	0	0	0	4
Morales, J., et al. (2021) [34]	1	0	0	0	0	1	0	1	0	0	0	1	0	1	0	5
Morales, J., et al. (2022) [35]	1	0	0	1	0	1	0	1	0	1	1	1	0	1	0	8
Nakamura, K., et al. (2021) [36]	1	0	0	1	0	1	0	0	0	1	1	1	0	1	0	7
Peric, D., et al. (2018) [37]	1	0	0	0	0	1	0	0	0	0	0	1	0	0	0	3
Pierantozzi, E., et al. (2022) [38]	1	0	0	1	0	1	0	0	0	1	1	1	0	1	0	7
Ramírez-Granizo, I. A., et al. (2020) [39]	1	0	0	0	0	1	0	1	0	0	0	1	0	0	0	4
Rocha et al. (2024) [13]	1	1	1	1	0	1	0	1	0	1	1	1	1	1	0	11
Suarez-Villadat et al. (2025) [14]	1	1	0	1	0	1	0	1	0	1	1	1	1	1	0	10
Toronjo-Hornillo, L., et al. (2018) [40]	1	0	0	0	0	1	0	0	0	0	0	1	0	0	0	3

Yes = 1/No = 0—1. Eligibility criteria specified; 2. Randomization specified; 3. Allocation concealment; 4. Groups similar at baseline; 5. Blinding of assessor (for at least one key outcome); 6. Outcome measures assessed in 85% of patients—6.1. adherence > 85%; 6.2. adverse events are reported; 6.3. exercise attendance is reported; 7. Intention-to-treat analysis; 8. Between-group statistical comparisons reported; 8.1. between-group statistical comparisons are reported for the primary outcome measure of interest; 8.2. between-group statistical comparisons are reported for at least one secondary outcome measure; 9. Point measures and measures of variability for all reported outcome measures; 10. Activity monitoring in control groups; 11. Relative exercise intensity remained constant; 12. Exercise volume and energy expenditure.

**Table 2:** Overview of the characteristics of the included studies.

Characteristic	Summary
Number of studies	27
Participants	1892
Primary population: ID	14
Judo	14
Duration	5–48 weeks
Frequency	1–3 sessions/week
Study design	RCT (4), QES (21), PS (1), RS (1)

**Table 3:** Detailed characteristics of the included studies.

Study	Sports	Population	Age Bracket (Years)	Sample Size	Program	Fall Prevention Teaching Program	Variables	Main Outcomes
Aguiar Jr, A. S., et al. (2008) [18]	Judo	Down Syndrome	Mean = 23.3 ± 2.1 (range not reported—NR)	*n* = 21	16 weeks—3 sessions per week (50 min)	-	Motor abilities, Biochemical markers	Exercise improved motor function and significantly decreased lactate production; Results reinforce the notion that physical exercise can improve motor disabilities and the increase of antioxidant defenses in people with Down Syndrome.
Arkkukangas, M., et al. (2020) [8]	Judo	Adult workers	Mean = 45 (18–68)	*n* = 79	10 weeks—1 session per week (50 min)—supervised judo-inspired exercise program at the respective workplace	Judo4Balance	Balance, Physical Fitness, Fall Quality	Adults can learn how to effectively breakfall in a controlled situation within a 10-week period; Results displayed equal benefits for men and women.
Arkkukangas, M., et al. (2021) [19]	Judo	Adult workers	Mean = 47 (18–68)	*n* = 142 (EG = 79, CG = 63)	10 weeks—1 session per week (50 min)—supervised judo-inspired exercise program at the respective workplace	Judo4Balance	Balance, Physical Fitness, Fall Quality	Implementation of the exercise program displayed significant differences in strength, balance, and safe falling techniques. The functions studied can effectively be trained in working-age adults by using the Judo4Balace exercise program.
Arkkukangas, M., et al. (2022) [9]	Judo	Older adults	Mean = 72 ± 4.9 (NR)	*n* = 200 (EG = 100, CG = 100)	12 weeks—1 session per week (50–60 min)—Judo4Balance program	Judo4Balance	Balance, Well-being, Physical Fitness, Fall Quality	The implementation of this exercise program resulted in significant improvements in physical function and in learning falling techniques.
Burschka, J. M., et al. (2014) [20]	Tai Chi	Multiple sclerosis	Mean = 43.13 ± 8.53 (NR)	*n* = 32 (EG = 15, CG = 17)	24 weeks—2 session per week (90 min)	-	Motor abilities, Balance; Well-being	Tai Chi may have beneficial effects on balance, coordination and psychological well-being in patients with MS.
Cha, J. Y., et al. (2020) [21]	Multisport	Autistic spectrum disorder and ID	Mean = 22.07± 2.55 (19–28)	*n* = 29 (EG = 15, CG = 14)	48 weeks—2 sessions per week (40–45 min)—Multisport Program: basic skills, floorball games, basketball and inline skating	-	Motor abilities, Physical Fitness	This study indicates that exercise programs could provide similar effects, even with other disorder types having similar symptoms.
da Silva, R. B. P., et al. (2022) [22]	MultiSport	Adults with visual impairment	Mean = 49.1 ± 8.7 (18–59)	*n* = 24 (EG = 12, CG = 12)	24 weeks [intervention period 1 (10 weeks); washout (4 weeks) and intervention period 2 (10 weeks)]	-	Well-being, Physical Fitness	The implementation of this exercise program resulted in significant improvements in physical function and in learning falling techniques.
DelCastillo-Andrés, O., et al. (2019) [23]	Judo	School-Age Children	Mean = 9 (6–12)	*n* = 453 (EG = 302, CG = 151)	6 weeks—Physical Education (PE) classes (10-min application of exercises aimed at teaching falls during warm-up)	Safe Fall	Fall Quality	Implementation of this program resulted in a considerable decrease in the risk of injury regardless of gender, BMI, academic year, and level of sports participation.
Du, J., et al. (2022) [24]	Tai Chi	Rheumatoid arthritis	Mean = 48.10 ± 5.55 (NR)	*n* = 20 (EG = 10, CG = 10)	12 weeks training program with selected Tai Chi movements and hand exercise	-	Mental Health; 4 Well-being	The selected tai chi movements and hand exercise is an effective treatment for Rheumatoid arthritis that improves joint pain, disease activity, quality of life, depression and anxiety.
Garcia, J. M., et al. (2020) [25]	Judo	Autism Spectrum Disorder	Mean = 12.31± 3.4 (8–17)	*n* = 14	8 weeks—1 session per week (45min)	-	Motor abilities	Preliminary results showed favorable increases in promote moderate-to-vigorous physical activity.
Ghadiri et al. (2022) [26]	Karate	Children with developmental coordination disorder	Mean—NR (12–13)	*n* = 16	8 weeks—3 sessions per week (75 min)	-	Motor abilities, Balance, Physical Fitness	Karate training serves as a good alternative for rehabilitation motor proficiency programs.
Gleser, J. M., et al. (1992) [27]	Judo	Blind, mentally retarded children	Mean—NR (6–12)	N = 7	24 weeks—2 sessions per week	-	Motor abilities, Mental Health	Reported improvements in physical fitness, motor skills, and psychosocial attitude.
Greco & De Ronzi (2020) [28]	Karate	Children with autism spectrum disorder	Mean = 9.25 ± 1.00 years (8–11)	*n* = 28 (EG = 14, CG = 14)	12 weeks—2 sessions per week (45 min).	-	Mental Health, Well-being	After intervention, children with autism spectrum disorder showed greater socio-emotional competence such as communication, cooperation and engagement, better executive functioning ability such as cognitive flexibility, inhibitory control and working memory and a lower aggressiveness, sadness, anxiety and hyperactivity.
Greenspan, A. I., et al. (2007) [29]	Tai Chi	Older adults	Mean—NR (≥70)	*n* = 269 (EG = 137, CG = 132)	48 weeks—2 sessions per week (90 min)	-	Well-being	Older women who are transitionally frail and participate in intensive Tai Chi exercise demonstrate perceived health status benefits, most notably in ambulation.
Kim, Y., et al. (2016) [30]	Taekwondo	Children with autism spectrum disorder	Mean = 10.14 ± 2.50 (6–15)	*n* = 14 (EG = 8, CG = 6)	8 weeks—2 sessions per week (50 min)	-	Balance	Children with autism spectrum disorder experienced improvements of postural control following participation in eight weeks of Taekwondo.
Li, Z. L., et al. (2019) [31]	Qigong (chi kung)	Parkinson’s disease	Mean—NR (40–80)	*n* = 126 (EG = 42, CG = 84)	12 weeks—3 sessions per week (60 min)	-	Motor abilities	Qigong exercise offers a potentially safe, low-cost and effective mind–body rehabilitative intervention for mitigating the problem of gait interruption among patients with Parkinson’s disease.
Lima, R. (2017) [32]	Capoeira	Deaf children and teenagers	Mean—NR (10–16)	*n* = 25	24 weeks—1 session per week (60 min)	-	Balance	It was possible to observe an improvement in the balance of the group who practiced capoeira, and consequently, a decrease in the risk of falling
Maslesa, S., et al. (2012) [33]	Judo	People With ID	Mean—NR (16–36)	*n* = 23	8 weeks—2 sessions per week (60 min)	-	Motor abilities	After the training process positive changes in motor abilities and motor skills have been noted.
Morales, J., et al. (2021) [34]	Judo	Children with Autism Spectrum Disorder	Mean—NR (9–13)	*n* = 11	8 weeks—1 sessions per week (75 min)	-	Motor abilities, Well-being	The study shows that participation in an adapted judo program helps to improve the motor skills and psychosocial behaviors of children with ASD
Morales, J., et al. (2022) [35]	Judo	Children with Autism Spectrum Disorder	Mean = 11.07 ± 1.73 (NR)	*n* = 40 (EG = 21, CG = 19)	24 weeks—1 session per week (90min)	-	Motor abilities, Mental Health	Implementation of this program resulted in improvements of the motor skills and psychosocial behavior of children with ASD.
Nakamura, K., et al. (2021) [36]	Judo	Patients with mental disorders	Mean = 41.0 years (20–64)	*n* = 34 (EG = 20, CG = 14)	24 weeks—2 sessions per month (60 min)	-	Well-being	Judo therapy enabled considerably fast improvement in the self-confidence and sense of accomplishment of patients.
Peric, D., et al. (2018) [37]	Karate	Mild ID	Mean = NR (16–19)	*n* = 15	12 weeks—3 sessions per week (60 min)	-	Motor abilities, Well-being	The karate program had a positive impact on the psycho-physical development of the examinees.
Pierantozzi, E., et al. (2022) [38]	Judo	Children with Autism Spectrum Disorder	Mean = 11.07 ± 1.73 (NR)	*n* = 40 (EG = 21, CG = 19)	24 weeks—1 session per month (90 min)	-	Motor abilities, Physical Fitness	The study showed that an adapted judo program for children with ASD can improve the cardio-metabolic health and cardiorespiratory fitness of its participants.
Ramírez-Granizo, I. A., et al. (2020) [39]	System of Oriental Struggles in Competition	Individuals with ID	Mean = 30.23 ± 9.854 (18–55)	*n* = 47	24 weeks—1 session per week (90 min)	-	Motor abilities, Balance	System of Oriental Struggles can be a motivational resource to adhere to healthy lifestyles and that also people with ID can have means for a significant improvement in their quality of life, helping to improve health status indicators (improvement in body mass index, strength level, flexibility, balance and coordination).
Rocha et al. (2024) [13]	Capoeira	People With ID	Mean = 15 ± 9 (NR)	*n* = 20 (EG = 10, CG = 10)	8 weeks—2 sessions per week (50 min)	-	Motor abilities, Balance	An intervention program with capoeira improves the psychomotor profile of people with ID.
Suarez-Villadat et al. (2025) [14]	Judo	Down Syndrome	Mean = NR (15–17)	*n* = 43 (EG = 24, CG = 19)	24 weeks—3 session per week (90 min)	-	Motor abilities, Balance, Well-being, Physical Fitness	Adapted judo significantly improved physical fitness components (strength, balance, and flexibility) in adolescents with Down syndrome. The intervention also enhanced psychosocial well-being, including self-esteem and social interaction.
Toronjo-Hornillo, L., et al. (2018) [40]	Judo	School-Age Children	Children Mean = 15.1 ± 2.45 (12–17)	*n* = 120	5 weeks—Physical Education (PE) classes (10-min application of exercises aimed at teaching falls during warm-up)	Safe Fall	Motor abilities, Fall Quality	The Safe Fall program responds proactively to the call from the WHO for the implementation of education programs based on fall-related research. It responds in this way to the overall objective of reducing the risk and severity of injuries produced by a sudden fall backward.

**Table 4:** Summary of the findings according to the main outcomes.

Outcome	No. Studies	Martial Arts	Population	Overall Findings
Motor abilities	13	Capoeira, Judo, Karate, Tai Chi, Qigong	ID, ASD, DCD, PD, Children, VI	Positive
Balance	12	Capoeira, Judo Karate, Taekwondo, Tai Chi	ID, ASD, DCD, MS, Deaf Children, Healthy populations	Positive
Mental Health	4	Judo, Karate, Tai Chi	ID, ASD, MS, RA	Positive
Well-being	8	Judo, Karate, Tai Chi	ID, DS, ASD, MS, RA, Healthy populations, VI	Positive
Physical fitness	8	Judo, Karate	ID, DS, ASD, DCD, VI, Healthy populations	Positive
Biochemical markers	1	Judo	DS	Limited evidence
Fall Quality	5	Judo	Healthy populations	Positive

## Data Availability

No new data were created or analyzed in this study. Data sharing is not applicable to this article.

## Supplementary Materials

The following supporting information can be downloaded at: https://www.mdpi.com/article/10.3390/healthcare14162644/s1, Supplementary Table S1: Complete search strategies for all electronic databases; Supplementary Table S2: Characteristics of structured judo-based fall-training programmes identified in the included studies.

## Abbreviations

The following abbreviations are used in this manuscript:

IDD	Intellectual and Developmental Disabilities
ID	Intellectual Disability
ASD	Autism Spectrum Disorder
DCD	Developmental Coordination Disorder
VI	Visual Impairment
DS	Down Syndrome
PD	Parkinson’s Disease
MS	Multiple Sclerosis
RA	Rheumatoid Arthritis
