# Supplementary material for: The Impact of Martial Arts-Based Fall Training Programs on Health and Well-Being in Individuals with Disabilities: A Systematic Review
Source: Healthcare (Basel). 2026 Aug 20;14(16):2644. doi: 10.3390/healthcare14162644 (PMC13512622; doi:10.3390/healthcare14162644)
Supplement: Supplementary file 1 [file healthcare-14-02644-s001.zip › healthcare-4411389-supplementary.pdf]

Supplementary Table S1. Complete search strategies for all electronic databases

Database	No database filters applied	Search Strategy (Equação de Pesquisa)	N
Web of Science	No database filters applied	("intellectual disabil*" OR "developmental disabil*" OR "learning disabil*" OR "cognitive impairment*" OR "people with disabil*" OR "special education need*" OR "special educational need*" OR "students with disabil*" OR "children with special need*" OR "neurodevelopmental disorder*")AND"judo"AND("exercise program*" OR "physical activit*" OR "physical education" OR "sport*" OR "fitness program*"OR "motor training" OR "movement training" OR "martial art*" OR "combat sport*" OR "karate"OR "taekwondo" OR "aikido" OR "jujitsu" OR "kung fu")AND("safe fall*" OR "fall prevention" OR "fall*" OR "balance" OR "postural control"OR "motor skill*" OR"coordination" OR "motor performance" OR "physical condition"OR "physical fitness" OR "injur*" OR "health" OR "wellbeing")	N=114
Scopus	No database filters applied	("intellectual disabil*" OR "developmental disabil*" OR "learning disabil*" OR "cognitive impairment*" OR "people with disabil*" OR "special education need*" OR "special educational need*" OR "students with disabil*" OR "children with special need*" OR "neurodevelopmental disorder*")AND"judo"AND("exercise program*" OR "physical activit*" OR "physical education" OR "sport*" OR "fitness program*"OR "motor training" OR "movement training" OR "martial art*" OR "combat sport*" OR "karate"OR "taekwondo" OR "aikido" OR "jujitsu" OR "kung fu")AND("safe fall*" OR "fall prevention" OR "fall*" OR "balance" OR "postural control"OR "motor skill*" OR"coordination" OR "motor performance" OR "physical condition"OR "physical fitness" OR "injur*" OR "health" OR "wellbeing")	N=15
Pubmed	No database filters applied	("Intellectual Disability"[Mesh] OR "Developmental Disabilities"[Mesh] OR "Learning Disabilities"[Mesh] OR "Cognitive Dysfunction"[Mesh] OR "intellectual disabil*"[tiab] OR "developmental disabil*"[tiab] OR "learning disabil*"[tiab] OR "cognitive impairment*"[tiab] OR "people with disabil*"[tiab] OR "special education need*"[tiab] OR "special educational need*"[tiab] OR "students with disabil*"[tiab] OR "children with special need*"[tiab] OR "neurodevelopmental disorder*"[tiab]) AND "judo"[tiab] AND ("Exercise Therapy"[Mesh] OR "Physical Fitness"[Mesh] OR "Sports"[Mesh] OR "Martial Arts"[Mesh] OR "exercise program*"[tiab] OR "physical activit*"[tiab] OR "physical education"[tiab] OR "sport*"[tiab] OR "fitness program*"[tiab] OR "motor training"[tiab] OR "movement training"[tiab] OR "martial art*"[tiab] OR "combat sport*"[tiab] OR "karate"[tiab] OR "taekwondo"[tiab] OR "aikido"[tiab] OR "jujitsu"[tiab] OR "kung fu"[tiab]) AND ("Accidental Falls"[Mesh] OR "Postural Balance"[Mesh] OR "Motor Skills"[Mesh] OR "safe fall*"[tiab] OR "fall prevention"[tiab] OR "fall*"[tiab] OR "balance"[tiab] OR "postural control"[tiab] OR "motor skill*"[tiab] OR "coordination"[tiab] OR "motor performance"[tiab] OR "physical condition"[tiab] OR "physical fitness"[tiab] OR "injur*"[tiab] OR "health"[tiab] OR "wellbeing"[tiab])	N=9
SPORTDiscus EBSCO	No database filters applied	TX(("intellectual disabil*" OR "developmental disabil*" OR "learning disabil*" OR "cognitive impairment*" OR "people with disabil*" OR "special education need*" OR "special educational need*" OR "students with disabil*" OR "children with special need*" OR "neurodevelopmental disorder*") AND "judo" AND ("exercise program*" OR "physical activit*" OR "physical education" OR "sport*" OR "fitness program*" OR "motor training" OR "movement training" OR "martial art*" OR "combat sport*" OR "karate" OR "taekwondo" OR "aikido" OR "jujitsu" OR "kung fu") AND ("safe fall*" OR "fall prevention" OR "fall*" OR "balance" OR "postural control" OR "motor skill*" OR "coordination" OR "motor performance" OR "physical condition" OR "physical fitness" OR "injur*" OR "health" OR "wellbeing"))	N=520

Supplementary Table S2. Characteristics of structured judo-based fall-training programmes identified in the included studies.

Programme	Associated studies	Population	Duration	Frequency	Main components	Outcomes evaluated
Judo4Balance	Arkkukangas et al. (2020, 2021, 2022)	Adult workers; older adults	10– 12 weeks	1 session/week	Breakfalls, balance, strength, coordination, high-challenge exercises	Balance, physical fitness, fall quality
Safe Fall	DelCastillo-Andrés et al. (2019); Toronjo-Hornillo et al. (2018)	School-age children; adolescents	5-6 sessions/week	1sessions/week 10-minute application of exercises aimed at teaching falls during warm-up	Backward falling techniques, motor responses, progressive practice	Balance, motor abilities, fall quality
